# Perceived fatigue in myotonic dystrophy type 1: a case-control study

**DOI:** 10.1186/s12883-019-1280-z

**Published:** 2019-03-28

**Authors:** Stefan Winblad, Christopher Lindberg

**Affiliations:** 1 Department of Psychology, University of Gothenburg, Box 500, 405 30 Gothenburg, Sweden; 2 Neuromuscular Centre, Department of Neurology, Sahlgrenska University Hospital, Gothenburg, Sweden; 3 Department of Clinical Genetics, Sahlgrenska University Hospital, Gothenburg, Sweden

**Keywords:** Myotonic dystrophy, Fatigue, Neuromuscular, Depression, Cognition

## Abstract

**Background:**

The aim of this study was to explore perceived fatigue, experienced functional limitations due to fatigue and clinical correlates in patients with Myotonic Dystrophy type 1 (DM1).

**Methods:**

In total, 32 consecutive patients with DM1 (14 women and 18 men) and 30 sex, age and education matched healthy control subjects participated. Perceived fatigue was rated on the Fatigue Impact Scale (FIS). Patients also completed a set of assessments aimed to characterize CTG-repeat size, muscle impairment, depression and cognitive functions. Non-parametric analysis were performed as appropriate, including Mann-Whitney U-test and Spearman correlation test.

**Results:**

DM1 patients had higher FIS total score than healthy controls, suggesting higher fatigue levels. More specifically, DM1 patients scored higher on the FIS physical and psychosocial subscales than controls but not on the FIS cognitive scale. Scores on fatigue correlated significantly with muscle impairment and depression.

**Conclusions:**

Perceived fatigue is significantly more common in patients with DM1 than in healthy controls. Higher ratings on depression and muscle impairment were associated with the condition. This indicates that both depression and muscle impairment may contribute to the experience of fatigue in DM1.

## Background

Myotonic dystrophy type 1 (DM1) is a slowly progressive inherited multisystem disorder, caused by the expansion of an unstable CTG trinucleotide repeat in the *DMPK* gene on chromosome 19q13.3 [1, 2]. The disorder negatively affects skeletal and smooth muscles, eyes, the heart and the endocrine- and central nervous systems. A high proportion of DM1 patients report fatigue [3]. Although a comprehensive and widely accepted definition is lacking, fatigue is commonly defined as an overwhelming sense of tiredness, lack of energy and feeling of exhaustion associated with difficulties in initiating or sustaining voluntary activities [4]. In many muscle dystrophies, muscle weakness and abnormal fatigability occurs after physical exertion [5]. This is the case in DM1; however, sleep disturbances and depression are also associated with the condition [6, 7]. Thus, in DM1 the cause of fatigue is multifactorial and may be associated with both central and peripheral nervous system abnormalities [8].

Since the first study on experienced fatigue in DM1 [9], several studies have explored the condition. These studies generally show a significantly higher prevalence of self-rated fatigue in patients with DM1, than as seen in normative data. However, to the best of our knowledge, only few case-control studies are available [10–14]. These studies have in most cases included small control samples, variable measures and with one exception [13] there is a general lack of information on effect sizes, making conclusions about the prevalence of fatigue, difficult to draw. Furthermore, few studies have presented information on the perceived impact of fatigue on different life domains. Available data indicate a positive correlation between higher fatigue levels and disrupted social participation (mobility, housing, employment and recreation) [15]. Patients scoring high on fatigue also describe reduced physical and social functioning, poorer mental health, less vitality and more physical pain. They also show lower levels of physical activity and less optimistic impression about their general health and quality of life [15, 16]. Few studies have explored fatigue and its relation to cognitive functions, except one that showed no correlation between fatigue and intelligence as measured on IQ tests [6].

The aim of the present study was to explore perceived fatigue in patients with DM1, using a standardized rating scale and to compare the patient’s results with those of matched healthy control subjects. We also analyzed whether patients age, disease duration, sex, degree of muscle impairment, CTG-repeat size, depression and performance on neuropsychological tests were associated with ratings on fatigue.

## Methods

### Subjects

Thirty-two patients (age range 23–61 years) with genetically confirmed adult-onset/classical DM1 [1] who consecutively attended the Neuromuscular Centre at the Sahlgrenska University hospital agreed to participate. The inclusion criterion was being 15 to 65 years old. In all cases, symptoms had first appeared after 10 years of age. All participants had normal or corrected to normal vision. Exclusion criteria included acquired brain injury, alcohol/drug misuse, and major psychiatric or somatic illness. No participant used psychostimulants during assessment. The control group consisted of 30 healthy individuals recruited from schools and workplaces in Gothenburg, matched with patients for sex, age and education. None of the control persons had any disease or was on any medication or drug. All participants gave informed consent and the regional ethical board in Gothenburg approved the study. Data on demographic and clinical characteristics are presented in Table 1. The subjects were recruited between 2010 and 2011.

**Table 1:** Demographic and clinical characteristics of participants^a^

	DM1 (*n* = 32)	HC (*n* = 30)	*p*-value
Age	40.1 (10); [39.5]	40 (11.1) [37.5]	.92
Proportion women	56.3%	53.3%	.82
Education, years	11.2 (2.3) [12]	11.6 (1.9) [12]	.96
Disease duration, years	16.2 (9.2) [15.5]		
CTG-repeat size^b^	712 (274) [750]		
MIRS	4.1 (1); [4.0]		
BDI-II	9.2 (6.3); [7.5]		

Note: *MIRS* = Muscular Impairment Rating Scale, *BDI* = Beck Depression Inventory, *HC* = healthy control subjects. ^a^Results are presented as mean (sd); [median]. ^b^*n* = 29

### Self-rating of fatigue

We used a Swedish version of the Fatigue Impact Scale (FIS), a self-rated questionnaire on fatigue with good psychometric properties [17, 18]. FIS includes 40 items, each of which is scored from 0 (no problem) to 4 (extreme problem), providing a continuous total score range from 0 to 160. High scores reflect functional limitation due to fatigue within the previous.

month in three different domains: physical (10 items), psychosocial (20 items) and cognitive (10 items). Physical functioning involves motivation, effort, coordination and stamina. Psychosocial functioning is associated with isolation, coping, workload and emotions. Cognitive functioning reflects memory, concentration, thinking and organizing one’s thoughts.

### Self-rating on depression

Beck Depression Inventory II (BDI II) was used to collect self-ratings of depression. BDI II is a widely used 21-item standardized self-report questionnaire measuring depression on a 4-point scale from 0 to 3 [19]. Proposed cut-off scores is as follows: 0–13, minimal depression; 14–19, mild depression; 20–28, moderate depression and 29–63, severe depression [19]. We also performed an item-analysis on two separable dimensions of depression: a cognitive affective (item 1, 3–14 and 17) and a somatic (item 15, 16, 18–20). The cognitive-affective dimension represents items such as lack of joy, guilt and suicidal thoughts and the somatic dimension includes a lack of energy, daytime sleepiness and exhaustion [19].

### Neuropsychological assessment

Patients with DM1 were given a standardized neuropsychological test battery measuring various cognitive functions. Tests included measured verbal ability (Vocabulary [20]), verbal fluency (F-A-S [21]), verbal memory (Rey Auditory Verbal Learning Test (RAVLT) [22]), visual construction ability (Rey Complex Figure Test (RCFT) [23]), visual memory (RCFT [23]), speed (Trail Making Test A (TMT A) [24] and Digit symbol [20]), attention (TMT A, B [24] and Digit Span [20]) and executive functions (Stroop Color Word Test B [25], TMT B [24] and Wisconsin Card Sorting Test [26]). The tests were presented in a previous study by the present authors [27].

### Procedure

An experienced neuropsychologist (the first author, SW) examined the patients at Sahlgrenska University hospital. The test procedure was performed during a two-hour session (with a break to avoid exhaustion) in the same order by all patients. A master student in clinical psychology under the supervision of SW performed the assessment on healthy controls at schools and workplaces. In all cases, participants completed testing in a quiet environment with adequate lighting.

### Rating on muscle impairment

Ratings on muscle impairments were performed by an experienced neurologist (CL) using the Muscular Impairment Rating Scale (MIRS) [28]. MIRS is an ordinal five-point rating scale, where 1 implies normal function/minimal symptoms and higher values indicates increasing levels of muscle impairment. MIRS 4 indicate mild to moderate proximal muscle weakness. The scale is a reliable measurement of muscular impairment in DM1 [28].

### Genetic analysis

DNA was extracted from peripheral blood lymphocytes and analyzed for the expansion of the CTG repeat in the DMPK gene. The analysis was performed using polymerase-chain-reaction and southern blot using the probe PM1 0 M6 [2]. The size of the CTG expansions was assessed visually from exposed x-ray films.

### Statistical analysis

Due to significant deviations from the normal distribution on several variables, non-parametric analysis were performed as appropriate, including Mann-Whitney U-test and Spearman correlation test with a Bonferroni-Holm correction for multiple tests [29]. We used Cohen’s guidelines [30] to interpret effect sizes as follows: small size <.30, medium effect size >.30 and large effect size >.50. The alpha level was set at .05. We compared total FIS score with normative data on Swedish control persons [31]. Data were analyzed using PASW base 18 (Chicago, IL).

## Results

**Fig. 1:**
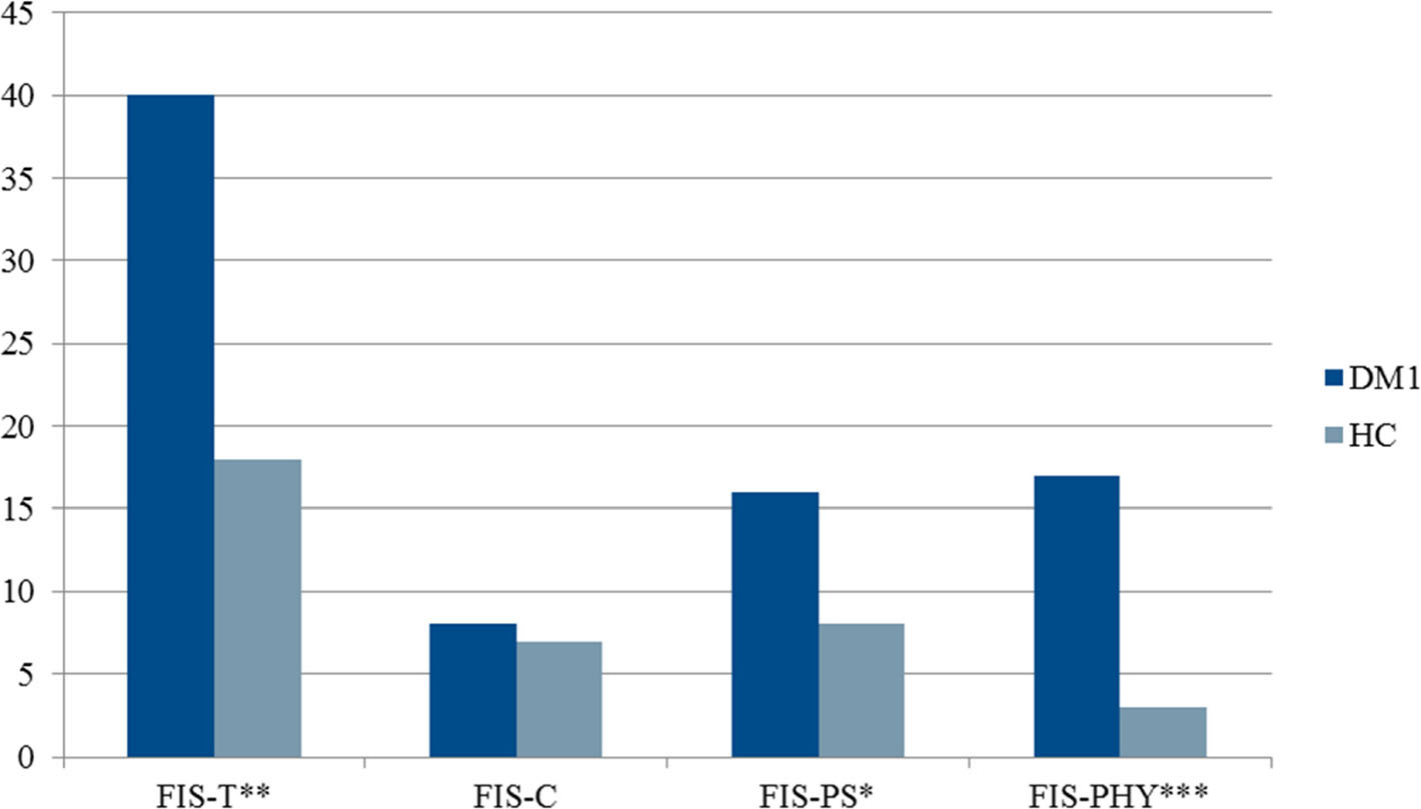
Median scores on FIS for patients with DM1 (*n* = 32) and healthy controls (*n* = 30). Note: FIS-T = total score, FIS-C = cognitive score, FIS-PS = psychosocial score, FIS-PHY = physical score, * = *p* < .05, ** = *p* < .01, *** = *p* < .001

Figure 1 shows FIS scores for the patients with DM1 and the healthy controls. A Mann-Whitney test for independent measures revealed significant between group differences in total FIS scores (*U* = 276, *p* = .004, *r* = −.37), FIS physical scores (*U* = 151.5, *p* = < .001, *r* = −.57), and FIS psychosocial scores (*U* = 318, *p* = .022, *r* = −.34) with medium to large effect sizes. With a total score of 44 used as a cut off (the 3rd quartile in a sample of 194 randomly selected Swedish control persons) [31] 50% of DM1 patients score above cut off while only 20% of the control sample did.

The correlation between DM1 patient’s scores on the FIS and depression, muscle function, disease duration and CTG repeat size was analyzed using Spearman’s correlation test. As shown in Table 2 there were positive correlations between scores on BDI-II and FIS total scores (r_s_ (30) = .68, *p* < .001), FIS cognitive scores (r_s_ (30) = .64, *p* < .001), FIS psychosocial scores (r_s_ (30) = .68, *p* < .001) and FIS physical scores (r_s_ (30) = .65, *p* < .001) with large effect sizes. We also analyzed the correlation between FIS and separate somatic and cognitive-affective BDI dimensions (see Methods section for details). Significant correlations (< .05) emerged between the two BDI dimensions, FIS total score and all FIS subscales. Muscle function as rated by MIRS correlated significantly with FIS total score (r_s_ (30) 0 .37, *p* < .05) and FIS psychosocial score (r_s_ (30) = .40, *p* < .05). To explore the association between perceived fatigue and results on the neuropsychological tests, we analyzed correlations between FIS scores and neuropsychological data (see Table 3). The FIS psychosocial scale correlated significantly with Trail Making Test A (r_s_ (30) = .37, *p* < .05). However, when corrected for mass significance using the Bonferroni-Holm test [29] with alpha set to .05, no significance remained. We found no differences between men and women in their ratings of fatigue, nor did we find any correlation between ratings of fatigue and duration of disease, age or CTG repeat expansion size.

**Table 2:** Correlations between ratings on fatigue and depression, muscular impairment, disease duration and CTG repeat size in patients with DM1 (*n* = 32)

	FIS-T	FIS-PS	FIS-PHY	FI-C
BDI-II	.68 **	.68**	.65**	.64 **
BDI-S	.43*	.41*	.47**	.46**
BDI-C	.49**	.50**	.42*	.46**
MIRS	.37*	.40 *	.34	.26
Disease duration (years)	.11	.14	.13	.10
CTG-repeats †	.06	.08	.09	−.11

Note: *FIS* = Fatigue Impact scale, *BDI* = Beck Depression Inventory, *MIRS* = Muscle Impairment Rating Scale. *FIS-T* = total score, *FIS-C* = cognitive score, *FIS-PS* = psychosocial score, *FIS-PHY* = physical score. *BDI-S* = BDI somatic dimension, *BDI-C* = BDI cognitive-affective dimension. * = *p* < .05, ** = *p* < .001, † = n = 29

**Table 3:** Correlations between ratings on fatigue and results on the neuropsychological assessment in patients with DM1 (*n* = 32)

	FIS-T	FIS-PS	FIS-PHY	FIS-C
Vocabulary	.17	.18	.08	.10
FAS	.00	−.03	.02	.01
RAVLT	−.01	−.04	.03	.04
RCFT copy	−.34	−.34	−.34	−.29
RCFT recall	−.30	−.32	−.28	−.23
TMT A	.32	.37*	.31	.08
TMT B	.20	.22	.20	.11
Digit symbol	−.06	−.12	.01	.02
Digit span	.05	.00	.06	.02
Stroop CWT B	.16	.17	−.22	.07
WCST (categories)	−.14	−.11	−.08	.17

Note: *FIS* = Fatigue Impact Scale, *FAS* = FAS Controlled Oral Word Association Test, *RCFT* = Rey Complex Figure Test, *RAVLT* = Rey Auditory Verbal Learning Test, *TMT* = Trail Making Test, *Stroop CWT* = Stroop Color Word Test, *WCST* = Wisconsin Card Sorting Test. *FIS-T* = total score, *FIS-C* = cognitive score, *FIS-PS* = psychosocial score, *FIS-PHY* = physical score. * = *p* < .05

## Discussion

Ratings of perceived fatigue were significantly higher in patients with DM1 than in matched healthy controls. This finding confirms significant between group differences [10–14]. We also found a moderate effect size when comparing FIS total scores. We found significant between group differences on the FIS psychosocial and physical domain with medium to large effect sizes. This indicates that more patients with DM1 experienced lower psychosocial functioning, workload and higher social isolation because of fatigue. These results are in line with an earlier report on disrupted social participation in DM1 [15]. More patients than controls also experienced limitations due to fatigue on psychological functioning, including emotional lability, irritability and stress and physical functioning, such as reduced motivation, poorer effort, and worse coordination and stamina. These results indicate that DM1 patients experience limitations due to fatigue, which is consistent with measurements of fatigue, psychological [6] and physical functioning [16]. Furthermore, there was a positive relationship between ratings on higher fatigue level and greater muscular impairment, which reinforces findings on fatigue and physical functioning [16].

Patients with higher scores on the FIS also scored higher on depression. Depression may be the result of general stress associated with disability in a chronic disease, where fatigue may be one factor leading to reduced activity, social isolation- and ultimately depression [32]. However, a depressive attitude may also negatively influence a person’s perception of their disability, including their perceived level of fatigue. When the association between perceived fatigue and two subdomains of the BDI II (a cognitive and a somatic) were analyzed we found that ratings on fatigue were related to both domains. This means that a depressive attitude may influence perceived fatigue, but also that fatigue may have an impact on ratings of depression. It is notable that although the ratings mainly showed signs of mild to moderate depression, only a few scores indicated severe depression, which replicates the results of an earlier study by our research group [33]. However, the correlation indicates that even small variations in experienced depression may influence fatigue, and vice versa.

We did not find any difference between groups regarding the FIS cognitive score and there were no significant correlations between perceived fatigue and any neuropsychological measure. These results complement earlier findings on fatigue and IQ [6] measured on demarcated cognitive functions. In other disorders, fatigue has been associated with higher physiological costs such as more widespread cerebral activation [34]. This physiological cost was related to the subjective experience of fatigue rather than to actual cognitive performance. Similarly, in DM1 fatigue and cognitive performance may operate through different mechanisms.

These findings are best understood in light of the study’s limitations. First, the assessment of fatigue were performed before the publication of an instrument specifically validated in patients with DM1 [35]. It is our impression that patients could understand and fill in the FIS satisfactorily, however the use of validated instruments are warranted in future studies. Second, excessive daytime sleepiness (EDS) is associated with DM1 [35]. Fatigue and EDS are strongly interrelated with muscle function and depression. Therefore, EDS may, indirectly influence correlations between fatigue, muscle function and depression. There was no rating on EDS in this study and it should be included in future studies as a covariate. Lastly, we did not find any correlation between ratings of perceived fatigue and measures of cognition. In other disorders [36, 37] ratings of fatigue have been associated in computerized tests over longer time interval with reduced attention and vigilance. The assessments in this study used only traditional paper-and-pencil tests over a shorter time span. We therefore propose that computerized tests should be included in future studies to increase sensitivity for detecting possible correlations.

## Conclusions

In summary, these data shows a significantly higher prevalence of perceived fatigue in patients with DM1 than in matched healthy control subjects, with an experienced impact on physical and psychosocial domains. These findings support the inclusion of fatigue as a main target for treatment interventions aimed to reduce fatigue through cognitive behavioral therapy and graded exercise. Such an intervention was recently applied and shown to reduce fatigue, increase activity and improve social participation [38].

## Abbreviations

BDI-II: Beck Depression Inventory II
DM1: Myotonic Dystrophy type 1
FIS: Fatigue Impact Scale
MIRS: Muscle Impairment Rating Scale
RAVLT: Rey Auditory Verbal Learning Test
RCFT: Rey Complex Figure Test
TMT: Trail Making Test
WCST: Wisconsin Card Sorting Test
